# A DNA-Based Biosensor Assay for the Kinetic Characterization of Ion-Dependent Aptamer Folding and Protein Binding

**DOI:** 10.3390/molecules24162877

**Published:** 2019-08-08

**Authors:** Irene Ponzo, Friederike M. Möller, Herwin Daub, Nena Matscheko

**Affiliations:** Dynamic Biosensors GmbH, Lochhamer Str. 15, 82152 Martinsried, Germany

**Keywords:** aptamer, G-quadruplex, thrombin, folding, kinetics, switchSENSE

## Abstract

Therapeutic and diagnostic nucleic acid aptamers are designed to bind tightly and specifically to their target. The combination of structural and kinetic analyses of aptamer interactions has gained increasing importance. Here, we present a fluorescence-based switchSENSE aptasensor for the detailed kinetic characterization of aptamer–analyte interaction and aptamer folding, employing the thrombin-binding aptamer (TBA) as a model system. Thrombin-binding aptamer folding into a G-quadruplex and its binding to thrombin strongly depend on the type and concentration of ions present in solution. We observed conformational changes induced by cations in real-time and determined the folding and unfolding kinetics of the aptamer. The aptamer’s affinity for K^+^ was found to be more than one order of magnitude higher than for other cations (K^+^ > NH_4_^+^ >> Na^+^ > Li^+^). The aptamer’s affinity to its protein target thrombin in the presence of different cations followed the same trend but differed by more than three orders of magnitude (K_D_ = 0.15 nM to 250 nM). While the stability (k_OFF_) of the thrombin–TBA complex was similar in all conditions, the cation type strongly influenced the association rate (k_ON_). These results demonstrated that protein–aptamer binding is intrinsically related to the correct aptamer fold and, hence, to the presence of stabilizing ions. Because fast binding kinetics with on-rates exceeding 10^8^ M^−1^s^−1^ can be quantified, and folding-related phenomena can be directly resolved, switchSENSE is a useful analytical tool for in-depth characterization of aptamer–ion and aptamer–protein interactions.

## 1. Introduction

Aptamers are single-stranded oligonucleotides with ideally high-binding affinity and specificity for their targets [1,2]. Currently, numerous aptamers have been selected for a wide range of targets, including proteins, small molecules, and even whole cells [3,4,5]. Due to the presence of some similarities and its many advantages, aptamers are often compared to antibodies, with similar hopes for diagnostic and therapeutic applications [6]. Both can be designed to bind their targets with very high affinities. The observable trend in the field of antibodies to develop bispecific or trispecific antibodies with two or more binding sites combined, applies to aptamers as well [7]. Multi-specific antibodies or aptamers mediate increased specificity, even slower off-rates from the target, or recruitment of specific effectors to a targeted site. However, multiple binding sites challenge the analysis of binding and unbinding rates, important factors in pharmacokinetics. Advantages of aptamers over antibodies start at the development stage. Raising a specific aptamer via an in vitro systematic evolution of ligands by exponential enrichment (SELEX) process takes less time in comparison to raising a new antibody, and does not have any animal requirements, which results in overall lower costs. During the very reproducible production it is easy to include site-specific chemical modifications or labels in aptamers. Nucleic acid aptamers exhibit a high thermal stability and good tissue penetration due to their small size [6]. Disadvantages of aptamers include their susceptibility to nucleases in the blood stream, which can be overcome by site-specific chemical modifications. For example, modifications of the 2′-OH of the nucleobase ribose, together with a 3′ inverted deoxythymidine (idT) cap, has led to an increased half-life in the blood stream of the first aptamer approved for clinical applications, the polyethylene glycol (PEG)-conjugated, anti-vascular epithelial growth factor (VEGF) aptamer NX1838 [8]. However, any modification introduced into an aptamer might change its affinity and specificity for its target as well, which needs to be carefully controlled.

A long-known and well-described example of a G-quadruplex aptamer is the 15mer thrombin-binding aptamer (TBA), recognizing the fibrinogen-recognition exosite I of thrombin via predominantly ionic interactions [9]. Thrombin is a serine protease and has a central role in the coagulation cascade. In normal conditions, thrombin is not present in human blood, but during coagulation its concentration varies from pM to µM levels [10,11]. Therefore, analytical assays with high sensitivity, but broad active range, for thrombin detection are of crucial importance in clinical practice. Numerous assays for thrombin detection have been developed up to date, with more than a hundred based on detection by aptamers alone [12]. All these assays come with strong differences in detection limits and have revealed a very broad range of dissociation constants (K_D_) for this aptamer–protein interaction, ranging from 200 pM [13] to 3 digit nM affinities [14]. The sensitivity of the interaction towards changes in the buffer system, assay orientation, detection method, and more, results in weak reproducibility of the K_D_. Nevertheless, the often-reported detection limit of lower pM thrombin concentrations could not be achieved with a nM K_D_, indicating that in, ideal conditions, thrombin is binding to TBA with a pM K_D_.

Moreover, historically, the TBA has been used as model system to demonstrate the proof-of-principle of aptamer-based assays in general [12]. Notably, single-molecule nanopore conductance studies have been performed to obtain data on label-free TBA folding and unfolding in the presence of different types of cations [15]. Considering the importance of the correct fold of the aptamer for its binding ability, it is of crucial importance to develop an easy assay that allows investigation of the role of solution components and target interaction on the aptamer fold. 

Since the binding site of aptamers is not the primary nucleic acid sequence, but the secondary structure, folding has a tremendous influence on the binding efficiency. Many aptamers that recognize biologically relevant protein targets reach activity by folding into G-quadruplex structures [16]. The DNA or RNA G-quadruplex structures have four guanines positioned in a square plane (G-tetrad) and have two or more G-tetrads stacked on top of each other [17]. The guanines coordinate a cation in the middle or in between G-tetrads, which is essential for correct folding. Interestingly, the type of cation is of great importance to the folding conformation. While both Na^+^ and K^+^ have been found to bind to the TBA, steady-state nuclear magnetic resonance (NMR) provided good indications that they do not form the same interactions with TBA and might induce alternative folds. NH_4_^+^, however, binds less stably than K^+^, but induces the same anti-parallel G-quadruplex fold [18]. The correct coordination of the cation among the two G-tetrads seems to depend on the ionic radius, since cations with ionic radii in the range of 1.3–1.5 Å fit well (K^+^: 1.33 Å, NH_4_^+^: 1.45 Å). In contrast, smaller or larger cations are not able to coordinate properly with the guanines. The Li^+^ and Na^+^ ionic radii are 0.6 Å and 0.95 Å, respectively, and therefore too small to fill the position of the central ion [15,19,20]. In addition to the induction of the folding of the G-quadruplex by ion coordination only, the role of target interaction is discussed as well. Structures of aptamers free of (apo) or bound by their target in a steady state have been solved by circular dichroism (CD) [14], crystal structures [21], and NMR [18]. These studies provide insight into the capability of different molecules to induce folding of aptamers which are not fully folded in their apo state (induced fit) [22]. Observation of the folding and unfolding processes in real-time, however, is limited by a scarcity of suitable methods. Commonly, fluorescence spectroscopy methods (e.g., fluorescence resonance energy transfer (FRET)) have been employed for the measurement of folding kinetics upon initiation by ions or target molecules. Even more challenging is the measurement of the unfolding rate, which requires fast wash-out of the analyte. This is not amenable in spectroscopy methods and to date mainly relies on modeling approaches. The application of standard methods for rate elucidation, notably surface plasmon resonance (SPR), to aptamer folding is limited. Both the detection of conformational changes, as well as binding of very small molecules such as ions to aptamers immobilized by large complexes, typically streptavidin, have proven difficult in SPR [23]. A better understanding of factors influencing folding and binding rates is necessary to gain control and build confidence in the sensitivity and specificity of aptamers.

Here, we introduce a convenient technology for the study of aptamers and their target interaction. switchSENSE (Dynamic Biosensors GmbH, Martinsried, DE) [24,25,26] is an emerging technology for biophysical quantification of binding and activity rates, in addition to a friction read-out. Adaptation of the DNA-based switchSENSE biosensors for aptamer measurements allowed us to profit from the inherent advantages of the technology for nucleic acid ligands, including quasi modification-free aptamer immobilization on the biosensor by hybridization. We characterized the well-described TBA model system by ranking K^+^, Na^+^, NH_4_^+^, and Li^+^ in their anti-parallel G-quadruplex folding capacity. We quantified the ion association (k_ON_) and dissociation (k_OFF_) rate and observed the folding and unfolding rates (k_F_, k_U_) in real-time. Furthermore, we report extraordinarily fast on-rates and dissociation constants (K_D_) down to the pM range for thrombin binding to TBA, depending on the buffer conditions. The broad dynamic range of detection of the aptasensor renders it a superior choice for quantification of thrombin from serum samples in diagnostics. Resolution of residence times and complex binding stoichiometries, on the other hand, are believed to aid in the development of therapeutics. Lastly, the resolved folding and binding processes allowed the conclusion of thrombin actively inducing the G-quadruplex fold.

In summary, targeting aptamers for diagnostics and therapy requires a detailed understanding of folding and interaction kinetics. We developed a powerful aptasensor for rapid and in-depth characterization of aptamers based on the switchSENSE technology, which can be easily adapted to various DNA/RNA aptamers.

## 2. Results

### 2.1. Development of the Aptasensor

**Figure 1:**
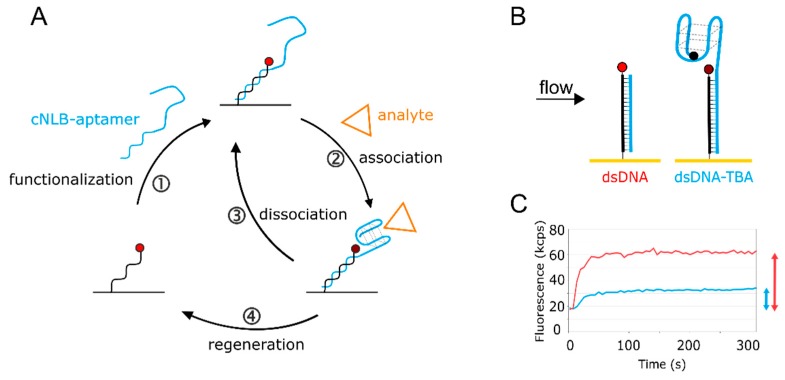
Aptasensor setup. (**A**) The aptasensor workflow consisting of ① sensor functionalization, ② + ③ analyte kinetics, and ④ sensor regeneration. The cycle can be automated to continue to bring various targets of interest onto the surface. (**B**) Scheme of the aptasensor surface. On the left, the immobilized single-stranded nanolever (NL) is functionalized with a complementary sequence (cNL), forming dsDNA. On the right, cNL is extended with the thrombin-binding aptamer (TBA) sequence, carrying a quencher on the distal end. The quencher is optional and enhances the signal. Without quencher, the 5′ guanine of TBA affects the fluorophore, but to a lower extent. (**C**) Real-time surface functionalization with dsDNA (red) and dsDNA-TBA with quencher (blue). Fluorescence amplitudes reached are marked with respective arrows.

Quantification of kinetic rates can be achieved on various commercial technologies. Since the switchSENSE biosensor comes with pre-immobilized DNA, it is designated for nucleic acid ligands. The general switchSENSE assay workflow is depicted in Figure 1A. We created an aptasensor by functionalizing the single-stranded (ss) DNA covalently immobilized on the switchSENSE biosensor (termed nanolever) via hybridization of a complementary strand (cNL-B) elongated with the aptamer sequence (Figure 1A, ①). The aptasensor setup can be seen in more detail in Figure 1B (right electrode). We extended the 5′ end of cNL-B with a short linker that is followed by the TBA sequence (see Material and Methods section for exact sequences). A quencher can be placed on the distal end of the aptamer sequence to enhance the folding read-out. Upon anti-parallel G-quadruplex formation, the quencher comes into proximity of the immobilized dye. Alternative conformations could be detected by placing the quencher at different positions on the aptamer, the resulting quenching efficiency will provide insight into the relative distance of the respectively targeted nucleotide to the dye. Here, the quencher can help to confirm aptamer immobilization on the surface. The hybridization of the complementary strand of the nanolever without TBA leads to the formation of a double-stranded (ds) DNA on the surface (Figure 1B, left electrode). Thereby, the fluorophore is pushed further away from the gold surface of the biosensor, since dsDNA is more rigid than ssDNA. This results in an increase of fluorescence due to the reduction in the quenching by the gold surface (Figure 1C, red trace). The hybridization of the TBA- and quencher-functionalized cNL-B leads to an increase in fluorescence as well, since the formation of the dsDNA base increases the distance of the dye from the gold surface. However, at the same time the quencher reduces the total fluorescence during the real-time functionalization of the sensor. Consequently, the fluorescence increase is smaller (Figure 1C, blue trace). The difference in fluorescence increase at full surface saturation (indicated by arrows on the right side of Figure 1C) allows to differentiate the biosensor functionalized with dsDNA from the aptasensor.

**Figure 2:**
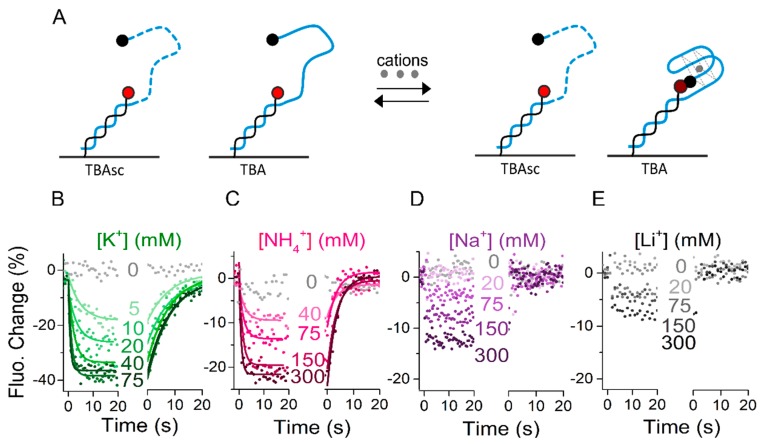
Kinetics of cation-dependent aptamer folding. (**A**) Schematic representation of the effect of cations on thrombin-binding aptamer (TBA) and scrambled TBA sequence (TBAsc) folding. Upon G-quadruplex formation the 5′ quencher is brought into proximity of the fluorophore, thus the fluorescence decreases. (**B**–**E**) Real-time fluorescence signal (dots) and fits (solid lines) of a representative experiment measuring the association and dissociation phases of: (**B**) K^+^; (**C**) NH4^+^; (**D**) Na^+^; (**E**) Li^+^ to immobilized TBA carried out in 50 mM Tris. Binding signal to TBA is referenced with binding signal to TBAsc.

**Figure 3:**
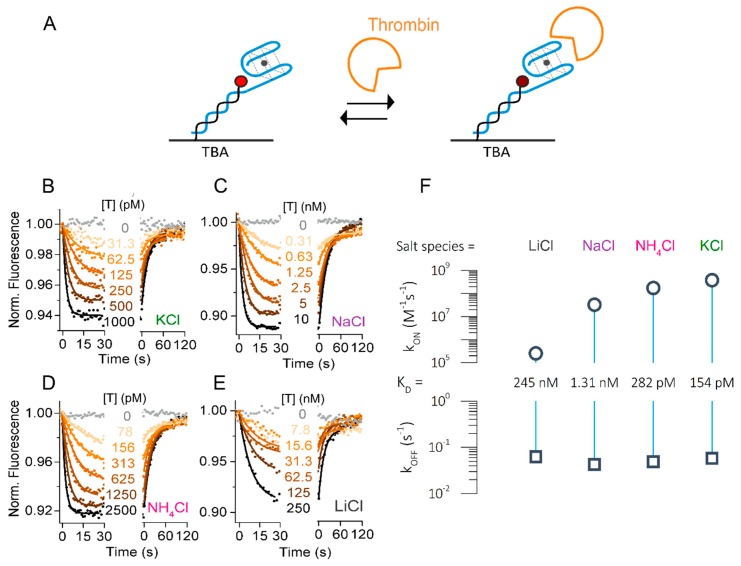
Influence of cation on thrombin–TBA kinetic rates. (**A**) Schematic representation of thrombin–TBA interaction. (**B**–**E**) Real-time fluorescence signal (dots) and fits (solid lines) of a representative experiment measuring the association and dissociation phases of thrombin (T) to immobilized TBA carried out in: (**B**) TE140-KCl; (**C**) TE140-NaCl; (**D**) TE140-NH4Cl; (**E**) TE140-LiCl. (**F**) Rate plot. The error bars (SD of three consecutively measured datasets) are smaller than the symbol size.

**Figure 4:**
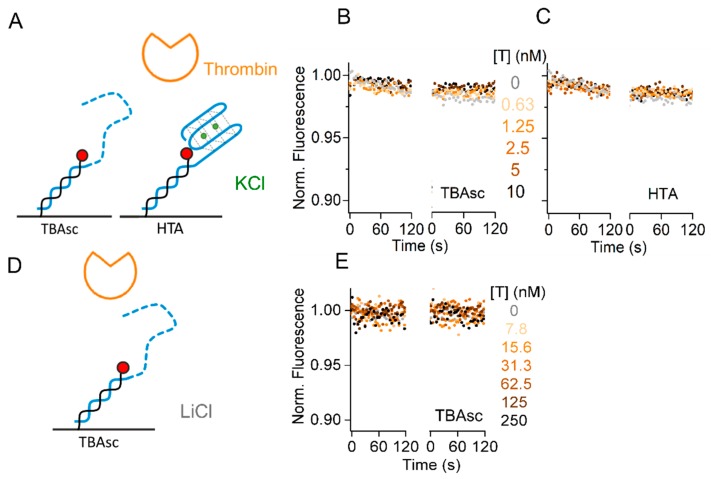
Specificity controls of thrombin–TBA interaction. (**A**,**D**) Schematic representation of the controls used. Real-time fluorescence signal (dots) during thrombin (T) kinetics with: (**B**) immobilized TBAsc; (**C**) immobilized human telomeric repeat aptamer (HTA) in TE140-KCl; (**E**) immobilized TBAsc in TE140-LiCl with indicated protein concentrations. In these conditions, no folding and/or binding were observed.

Once the functionalization of the surface with TBA has been confirmed, the ion or protein analyte is injected. Folding and binding kinetics can be measured in real-time upon association and washing out of analyte, respectively. The analyte influences the local fluorophore environment (see Supplementary Materials Section S2 for switchSENSE FPS mode), leading to a fluorescence change upon binding (Figure 1A, ②) and restoring the original fluorescence level upon removal (Figure 1A, ③). Washing the biosensor surface with regeneration solution allows to remove any hybridized ligand and prepares the immobilized ss NL for the injection of the next sample (Figure 1A, ④). This automated workflow enables sampling of various targets of interest with low sample consumption.

### 2.2. Real-Time Kinetics of Aptamer Folding

As discussed in the introduction, cations influence the formation and stability of the G-quadruplex structure. However, rates of ion-induced folding and unfolding have only been reported in sporadic studies [15]. Therefore, we first subjected the immobilized aptamers to K^+^, NH_4_^+^, Na^+^, and Li^+^ ions and compared their influence on the G-quadruplex folding. Employing the setup shown in Figure 1A, the association of ions to TBA was observed by a concentration-dependent fluorescence decrease. The aptamer sequences were functionalized with a quencher (BBQ-650^®^) at the 5′ end for enhanced signal amplitude for these experiments. Importantly, the microfluidic setup was able to wash out the ions tested on an ms timescale, allowing the aptamers to release bound cations and come back to an unfolded state. Correspondingly, dissociation of ions resulted in a fluorescence increase back to starting levels, representing the unfolding. Addition of salt as analyte changes the ionic strength of the solution and might have an influence on the fluorophore itself. To correct for the influence of the ionic strength on the fluorescence signal, data shown in Figure 2 are referenced with the signals obtained by injection of the same analyte solutions to a non-folding control, the “scrambled TBA” (TBAsc). It contains the same nucleotides as TBA, but in a changed order that prevents G-quadruplex formation (Figure 2A). Figure 2B–E shows the real-time concentration-dependent folding in the presence of K^+^, NH_4_^+^, Na^+^, and Li^+^.

We observed a strong signal decrease upon injection of K^+^, related to an efficient ion binding and, thus, aptamer folding (Figure 2B). Fitting the K^+^ kinetics yielded a K_D_ of 8 mM (k_ON_ and k_OFF_ rates are reported in Table 1). We observed a similar behavior for NH_4_^+^ ions (Figure 2C), though with slower on and faster off rates and a K_D_ of 104 mM. The dependence of the conformational change on ion concentration indicated that the rate-limiting step was ion binding and not folding. At highest ion concentration, the ion binding was not rate-limiting anymore, therefore we interpreted the observed rate as the maximum possible folding rate (see the Discussion section). We observed TBA folding at a rate of 1.26 s^−1^ in the presence of 75 mM KCl, and only slightly slower at 1.18 s^−1^ in the presence of 300 mM NH_4_^+^. Interestingly though, in both KCl and NH_4_Cl we got close to the maximum saturation levels (amplitudes levelling off at minimum), the total fluorescence change was much larger in KCl (~40%) than in NH_4_Cl (~25%). Since the extent of quenching was directly related to the distance of the quencher from the dye, a lower amplitude represents an average larger distance. It should be noted that the differences in amplitude can be caused by less ion binding/folding and/or an alternative folding state. Association and dissociation of Na^+^ and Li^+^ ions resulted in concentration-dependent step function signal decreases and increases, respectively, which could not be fit with a mono-exponential fit function (Figure 2D,E). This indicates very low affinity of these ions to the aptamer or low antiparallel G-quadruplex folding capacity. Our findings correspond with Li^+^ generally being regarded as a monovalent cation having no effect on G-quadruplex folding [15,27]. The slight decrease in fluorescence signal upon Li^+^ injection was likely caused by non-G-quadruplex like conformational changes that alter the distance of the quencher to the dye. Similarly, Na^+^ has been reported to induce folding of an alternative conformation, which is more dynamic and does not end with the 5′ quencher in close proximity of the dye [18].

Overall, our results show G-quadruplex folding induced by K^+^ and, with lower affinity for TBA, by NH_4_^+^. Na^+^ was shown to have a very low affinity and likely induces alternative conformations, while hardly any binding of Li^+^ was observed.

### 2.3. Influence of Salt Species on Thrombin-Binding Kinetics

Analysis of the folding and unfolding rates and extent of TBA folding showed a clear dependence on the nature of the ion present in solution. The effect of pre-folded G-quadruplex on thrombin binding was not clear. Therefore, we next determined the influence of the different ions on the kinetic rates of thrombin binding to TBA (Figure 3A). All ions were used at a fixed concentration of 140 mM. In contrast to the folding experiments, we did not functionalize the aptamer with a quencher for these binding experiments, because fluorescence signal changes due to the changes in the local environment of the immobilized dye were observed upon thrombin binding even without quencher. Both the protein itself, as well as the 5′ terminal guanine of TBA, are able to affect the fluorescence.

Figure 3B–E shows the real-time concentration-dependent association and dissociation curves of thrombin interaction in the presence of K^+^, NH_4_^+^, Na^+^, and Li^+^. In this case, the association of thrombin to TBA was detected by a concentration-dependent fluorescence decrease, and its dissociation resulted in a fluorescence increase back to initial levels. Single-exponential binding behavior was observed as expected for a one-to-one-interaction. Global fitting of the curves yielded on-rates strongly dependent on the nature of the ions in solution. Surprisingly, K^+^ enabled an extraordinarily fast on-rate of k_ON_ = 3.8 × 10^8^ M^−1^s^−1^. At this point, we would like to highlight that these extremely fast on-rates can only be resolved with sufficiently high flow rates. Supplementary Materials Figure S1 shows the effect on k_ON_ and k_OFF_ in the presence of K^+^ at 100, 500, and the applied 2000 µL/min flow rates. Slower analyte transport yielded lower on-rates due to the mass transport limitations, as well as reduced off-rates due to the rebinding. 

Comparing the two physiologically relevant ions K^+^ and Na^+^, we found that k_ON_ of thrombin in the presence of Na^+^ was 10 times lower than in the presence of K^+^ (k_ON_ = 3.3 × 10^7^ M^−1^s^−1^, Figure 3C). This is in good agreement with the folding data and literature: K^+^ ions stabilize the folding of TBA into a G-quadruplex and thus facilitate thrombin binding [28,29]. In contrast, alternative conformations in the presence of Na^+^ [18] first need to be transformed to the thrombin-binding anti-parallel G-quadruplex fold. Furthermore, we analyzed the kinetics in the presence of ammonium ions, which have been reported to pre-fold the same G-quadruplex as K^+^, though with lower affinity [18]. The previously determined K_D_ of NH_4_^+^ results in a bit more than 50% of TBA being pre-folded in the applied condition of 140 mM salt. Interestingly, we found that the affinity of thrombin was in the same range as in the presence of K^+^, with the k_ON_ rate reduced by a factor of 2 (1.7 × 10^8^ M^−1^s^−1^, Figure 3D). 

The effect of the presence of Li^+^ ions is controversial, but it is considered to be the least efficient in folding the active G-quadruplex [19]. Nevertheless, we observed concentration-dependent association of thrombin to TBA in LiCl buffer. Here, the on-rate of thrombin was significantly lower at 2.6 × 10^5^ M^−1^s^−1^, which is 1000 times slower than in the presence of K^+^. Accordingly, the interaction was observed only in the presence of comparably high thrombin concentrations above 7 nM (Figure 3E). Intriguingly, the ion-dependent shift in affinity was solely caused by changes in the on-rate, while the off-rate was well comparable in all conditions tested. This results in K_D_s in the picomolar to nanomolar range, as listed in Table 2 and visualized in the rate plot in Figure 3F. 

Taken together, we showed the dependence of thrombin association on the type of cation in solution and resolved on-rates of thrombin to TBA up to the 10^8^ M^−1^s^−1^ range in best conditions, which contain cations that facilitate thrombin binding by pre-folding of the G-quadruplex.

### 2.4. Specificity of TBA for Thrombin Interaction Allows Broad Dynamic Range

We showed the influence of the cation species on the TBA–thrombin kinetics, applying thrombin concentrations from 31 pM to 250 nM to reach significant fractions bound in all buffers. Many methods are limited in their dynamic range, by lacking sensitivity for low concentrations and increasing the background at high concentrations. The challenging detection of low pM concentrations can be achieved by the switchSENSE aptasensor within 30 s contact time (Figure 3B). Next, we designed specificity controls to exclude background contributing to our binding signals at higher protein concentrations. To study the sequence and structure specificity of the thrombin–aptamer interaction, we used two control sequences. First, the TBAsc which does not fold into a G-quadruplex structure. Second, the Human Telomeric repeat Aptamer (HTA), which is another G-quadruplex forming aptamer. Both aptamers were tested in the best binding conditions (TE140-KCl) with a thrombin concentration of up to 10 nM, covering the range of concentrations applied in K^+^, NH_4_^+^, and Na^+^ buffers. As shown in Figure 4A, we could not observe any association of thrombin to TBAsc, indicating that our obtained binding signal in Figure 3 was specific for the folded TBA structure. Furthermore, we neither observed thrombin binding to HTA (Figure 4B), which is known to form a G-quadruplex structure in the presence of K^+^ [30]. These findings underline not only the structure- but also sequence-specificity of the thrombin binding aptamer.

Lastly, we increased the protein concentration to 250 nM in TE140-LiCl and checked for non-specific interactions of this high protein concentration with TBAsc. The lack of signal in Figure 4C confirms that even at high concentrations, thrombin binding is specific at the salt concentration applied for the binding experiments. In conclusion, we showed a broad dynamic range of the aptasensor from low pM to high nM target concentrations.

Importantly, we maintained an ionic strength of 150 mM in all tested conditions and only altered the type of cation added to the buffer. This was a prerequisite for specific thrombin interaction, since in a background of 50 mM Tris without any additional salt, we observed non-specific interaction of thrombin with dsDNA at high protein concentrations (>100 nM) (Supplementary Materials Figure S2C). The presence of TBA or TBAsc in this no-salt condition reduced the minimum thrombin concentration leading to a non-specific signal to 62.5 nM thrombin, with an overall higher signal amplitude (Supplementary Materials Figure S2A,B). These controls indicate that low ionic strength can lead to non-specific interaction of thrombin with ds and ssDNA.

Taken together, we recommend always including well-designed control aptamers to confirm binding specificity in the chosen concentrations and buffer conditions. Automated sensor functionalization with various aptamers in the switchSENSE technology supports fast screening of conditions.

### 2.5. Immobilized Target for Aptamer Screening

**Figure 5:**
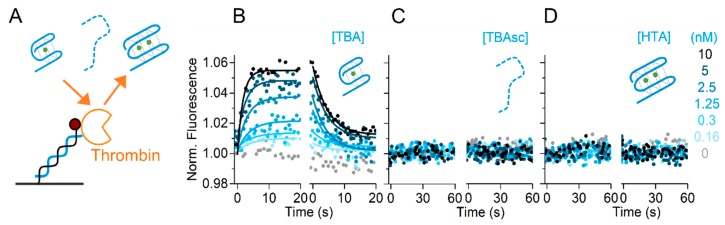
Reversed assay orientation. (**A**) Schematic representation. (**B**–**D**) Real-time fluorescence signal (dots) and fits (solid lines) during association and dissociation phases of aptamers to immobilized thrombin carried out in TE140-KCl. Thrombin binding kinetics of (**B**) TBA; (**C**) TBAsc; (**D**) HTA. In (**C**) and (**D**) no binding was observed.

All data shown so far were obtained with the aptamers immobilized on the sensor surface. However, the process of aptamer selection SELEX [1,2] yields a variety of different aptamers, which need to be screened against one target. Next, we show a reversed assay format that facilitates such screenings of the obtained aptamers. In this setup, thrombin was immobilized on the sensor surface as covalent conjugate to the cNL-B sequence. The same measurement workflow as described in Figure 1 was applied for the immobilization of thrombin on the sensor. In the first step, the DNA conjugated to thrombin was hybridized to the surface-immobilized ssDNA nanolever. Subsequently, the thereby thrombin-functionalized surface was exposed to different aptamers (TBA, TBAsc, HTA) in TE140-KCl buffer, as indicated in Figure 5A. Association of TBA to thrombin was detected by a concentration-dependent fluorescence increase, and its dissociation resulted in a fluorescence decrease back to initial levels. Figure 5B–D shows the real-time concentration-dependent association and dissociation data of TBA, TBAsc, and HTA with thrombin. We confirmed specific binding of TBA to immobilized thrombin (Figure 5B). The resulting association and dissociation rates were k_ON_ = 4.6 ± 1.9 × 10^7^ M^−1^s^−1^ and k_OFF_ = 2.8 ± 0.6 × 10^−1^ s^−1^, respectively, yielding a K_D_ = 6.2 ± 4 nM. It is noteworthy that the dissociation constant was higher than determined in the previous assay orientation. Nonetheless, the screening correctly identified TBA as the binding partner. In contrast, neither TBAsc nor HTA showed any interaction (Figure 5C,D). These controls confirm that the immobilized thrombin was only recognized by specifically raised aptamers. The possibility to reverse the assay orientation proves the versatility of the switchSENSE technology for aptamer studies and broadens the field of applications.

## 3. Discussion

The purpose of this study was to facilitate high-quality, in-depth analysis of aptamers. We demonstrate how the switchSENSE technology can resolve aptamer folding and unfolding upon interaction with small molecules, such as ions, and provide rates of target kinetics. We chose thrombin and its well-described 15mer TBA [9] as a model system to develop an aptasensor and investigate advantages and limitations of the switchSENSE technology for aptamer measurements.

### 3.1. Real-Time Folding and Unfolding Rates Coupled to Ion Binding

The switchSENSE technology measures the response of a surface-immobilized fluorophore to changes in its local surrounding induced by injection of analytes on the ms timescale. Thus, it allows to observe the influence of both the injection and wash out of ions on the TBA conformation in real time and to deduce the rate of folding (k_F_) and unfolding (k_U_). The conformational change in TBA was directly dependent on the cation binding to the aptamer. The association of positively charged cations to the negatively charged DNA was strongly driven by ionic interaction and affected the DNA self-interaction. Furthermore, fluorophores were sensitive to ionic strength. This resulted in control DNA TBAsc showing the same concentration-dependent, but cation type-independent, signal change in response to all cations (see raw data in Supplementary Materials Figure S4). In contrast, TBA reacted in a cation-dependent way beyond the signal of ion binding alone. To extract the TBA-specific rates, we therefore referenced the TBA with TBAsc signal. 

This leaves us with a rate that had in fact two components: 1) the kinetics of the ion with TBA; and 2) the rate of the conformational switch in response to the ion. Though we observed some degree of folding in response to all cations tested, only in the presence of K^+^ and NH_4_^+^ could we apply a global mono-exponential fit and observe concentration-dependent signal saturation. The dependence of the folding rate on the ion concentration indicated that the ion-binding was the rate-limiting step at lower concentrations. The observed rate constant, therefore, represents k_ON_ of the cation. We report k_ON_ of K^+^ = 15.2 M^−1^s^−1^ and k_ON_ of NH_4_^+^ = 2.96 M^−1^s^−1^. Though unfolding dynamics cannot be synonymized with ion residence time [19], the ion must dissociate from the complex to allow unfolding. By the example of NH_4_^+^, this is allowed by an exchange rate from TBA-bound to bulk of 1.0 s^−1^ at 15 °C [18]. This rate is very close to the unfolding rate in our setup k_U_(NH_4_^+^) = 0.31 s^−1^, measured at 25 °C. In contrast to binding, it is not possible to limit the unfolding rate by changing the ion concentration. We, therefore, used TBA unfolding rates synonymously to the minimal ion dissociation rates. In line with literature, K^+^ not only showed faster on-rates, but also slower off-rates (k_U_(K^+^) = 0.12 s^−1^) than NH_4_^+^. Both Na^+^ and Li^+^ exhibited residence lifetimes below our ms resolution, which indicates that they were inducing rapidly, switching alternative conformations.

We have concluded that sub-maximum ion concentrations limit the folding rate. Conversely, applying ion concentrations above their binding saturation level should result in maximum folding rates. We, therefore, report a K^+^-dependent observed folding rate of 1.26 s^−1^ in a background of 75 mM KCl. NH_4_^+^ induced slower folding at 1.18 s^−1^ at 300 mM. It should be noted that this rate was still limited by ion concentration, since the K_D_ = 104 mM indicates NH_4_Cl should be used at ~1 M to reach saturation. Measured by an entirely different method, Shim et al. [15] obtained very comparable folding kinetics of TBA, particularly the unfolding rate (k_U_(K^+^) = 0.07 s^−1^; k_U_(NH_4_^+^) = 0.25 s^−1^). The employed method of single-molecule nanopore conductance studies directly measures the unfolding rate k_U_. However, the observed folding rate k_F_ was calculated from K_F_ = k_F_/k_U_. In contrast, here we observed both folding and unfolding rate directly. 

The K_D_ of potassium (K_D_ = 8 mM) was more than one magnitude higher than for NH_4_^+^, while folding by Na^+^ and Li^+^ could not be quantified. The difference in folding capacity has been related to the ionic radius (see the Introduction). Na^+^ is too small to properly coordinate with the guanines of the G-tetrad. Therefore, TBA mainly folds into a parallel-type G-quadruplex structure in the presence of Na^+^ ions [18], which did not result in efficient quenching in our setup. Correspondingly, we could not quantify folding rates in the presence of Na^+^ and Li^+^. Nevertheless, the amplitudes reached at a fixed ion concentration, representing the fraction folded, confirmed the order of the folding capacity to be K^+^ > NH_4_^+^ > Na^+^ > Li^+^ (Supplementary Materials Figure S3A), as suggested by the literature [31,32]. 

Due to the surface immobilization by a complementary sequence, the total DNA oligo studied here differs from the label-free TBA sequence used in solution assays. The TBA sequence was originally identified as consensus sequence from several longer DNA oligos interacting with thrombin [9]. This indicates that TBA is able to form the thrombin binding site even in the presence of additional ssDNA. The good agreement of folding rates determined by our method and by the label-free conductance study indicates that the immobilization of aptamers via extended DNA does not alter folding rates significantly. This is not surprising, since the additional DNA is rendered dsDNA by hybridization to the surface and is therefore removed from the folding reaction. Consequently, we consider TBA in our setup to be quasi label free.

### 3.2. Resolution of High-Affinity TBA–Protein Interaction

As in the folding experiments, the kinetic rates of thrombin binding showed a strong dependence on the applied flow rate (Supplementary Materials Figure S1). This indicates that artefacts such as mass-transport limitation during the association and rebinding during the dissociation are easily distorting measured TBA–thrombin kinetic rates. To minimize these technical limitations, all folding and binding experiments were performed at a high flow rate of 2000 µL/min and a low surface density. One electrode can be functionalized with approximately 200 TBA molecules/µm^2^ (equivalent to ≈ 10 RU in SPR), which results in roughly 2 × 10^6^ molecules per electrode. Profiting from the high-performance microfluidic system, we were able to resolve thrombin binding to TBA at unprecedented rates. Impressive is the on-rate of 3.8 × 10^8^ M^−1^s^−1^ in the background of KCl, considering how close this is to the theoretical diffusion-limited on-rate in the range of ~10^9^ M^−1^s^−1^. In line with literature, K^+^-based buffers are the most efficient in promoting thrombin binding [15,33,34]. The corresponding dissociation constant K_D_ = 154 pM compares well with the lowest values reported in the literature of 200 pM [13]. Replacement of K^+^ by other cations, while keeping the ionic strength constant, reduced the on-rate down to 2.6 × 10^5^ M^−1^s^−1^, resulting in a K_D_ = 245 nM (LiCl buffer). Situated in between is the low nM K_D_ in NaCl-based buffer, which compares well with K_D_ = 7.1 nM determined in similar conditions by SPR [35]. Interestingly, the off-rate was rather independent of the cation present and remained at ~5 × 10^−2^ in all tested buffers.

The developed aptasensor is a high-sensitivity assay with a detection limit of <30 pM in TE140-KCl (Figure 3B). Even lower concentrations of thrombin have been reported in the literature, e.g., 10 pM were measured by cationic polythiophene derivatives, which produce a fluorometric read-out [36]. These detection limits fit with a K_D_ in the pM range, since a nM K_D_ would not result in any significant surface saturation at low pM concentrations. Importantly, the low pM detection limit does not prevent the application of high nM concentrations of thrombin to the aptasensor.

In conclusion, for the first time, we can report TBA–thrombin rates resulting in a K_D_ of 150 pM, explaining the high sensitivity of many existing assays.

### 3.3. Differentiation of Prefolded Aptamer from Induced Fit

Although Li^+^ and Na^+^ ions do not promote TBA to fold into a G-quadruplex, specific thrombin binding was observed at high concentrations anyways. Though surprising at first, this finding is in line with the literature reporting thrombin binding even in the absence of salts [22,37,38]. Though our controls (Supplementary Materials Figure S2) caution that binding of thrombin at low salt might be strongly non-specific, well-referenced studies can conclude binding due to the induced fit. Here, thrombin-binding experiments were performed at a fixed cation concentration of 140 mM in the buffer. In the case of K^+^, which was shown to have a K_D_ of 8 mM in Figure 2, this led to the majority of TBA on the chip being folded. In contrast, NH_4_^+^, Na^+^, and Li^+^ at the same concentration showed significantly less signal quenching, i.e., G-quadruplex formation (Supplementary Materials Figure S3A). Due to the lower thrombin affinity, higher concentrations of thrombin had to be applied in these buffers to reach comparable surface saturation in the background of NH_4_^+^ and Na^+^. In the presence of Li^+^, even with 250 nM of thrombin, only about 50% saturation was reached (Supplementary Materials Figure S3B). The surface saturation at specific protein concentrations can be calculated as fraction bound (%) from the measured kinetic rates (Supplementary Materials Figure S3D–G). Interestingly, though the thrombin binding rate was the highest in presence of K^+^, this condition shows the smallest signal amplitude (6%) (Figure 2 and Supplementary Materials Figure S3C). Though saturating a comparable fraction of TBA, both NH_4_^+^ and more so Na^+^, induced larger signal changes (8% and 11%, Figure 2 and Supplementary Materials Figure S3C). Even in the presence of Li^+^, when only half of the TBAs were saturated with thrombin, the quenching effect was larger than in K^+^ and NH_4_^+^ (9%). The comparison of the signal amplitudes from folding and binding suggests that the higher the fraction of aptamers already correctly folded by the ion alone, the smaller the signal change upon thrombin binding. Therefore, the thrombin binding assay was not only detecting the protein itself, but the induction of G-quadruplex folding. This is feasible even without quencher, since the guanine on the 5′ end has quenching properties itself [39]. These results corroborate previous findings that thrombin binding induces G-quadruplex conformation in those conditions that have not induced the correct fold before target association [33].

Another hint at folding induction by thrombin binding can be found in the protein off-rate. Considering that the off-rate of thrombin is the same in all buffers, it is likely that the induced fit creates a similar binding site in all conditions tested. This corresponds to crystal structures of the TBA–thrombin complexes in the presence of K^+^ and Na^+^, both of which have a very similar TBA structure [21]. This is in contrast to TBA without thrombin, which exhibits alternative conformations in the presence of the abovementioned cations [18].

Taken together, our folding studies provide clear evidence for folding of the G-quadruplex depending on the type and concentration of the cation present. Combined with the thrombin interaction studies, we can conclude that thrombin further forces unfolded TBA into the correct G-quadruplex structure. Therefore, our data confirm the induced fit model of TBA–thrombin binding.

### 3.4. Contribution of Results to Therapeutic and Diagnostic Applications

One important application of aptamers is in diagnostic applications. These require detection limits low enough to cover all naturally relevant concentrations. Moreover, minimal sample processing prior to measurements is an advantage. By the example of thrombin, the range of free thrombin varies between low pM and µM concentrations in the blood and indicate the level of thrombosis risk [10,11]. Many aptamer-dependent assays have been developed with pM sensitivity, while others only detect thrombin at a nM concentration with the same probe and target. The detection limit is directly correlated with the interaction affinity. We show a clear dependency of the TBA–thrombin affinity on the type of cation present. Due to the ionic nature of the interaction it is not surprising that an increase in salt concentration results in a decrease of the binding affinity [14]. Here, we kept the ionic strength of the buffer constant and only changed the type of cation present. This simple buffer adaptation decreased the K_D_ from 1.3 nM in the presence of NaCl to 154 pM in the presence of KCl. Since many assays have been developed with NaCl-based buffers, they might be further improved by comparable alterations to the buffer composition. Another advantage of the switchSENSE technology, though not shown here with the applied in vitro samples, is the possibility to directly inject up to 80% serum samples onto the sensor (according compatibility sheet by Dynamic Biosensors GmbH [40]). This paves the way for a highly sensitive diagnostic application of the aptasensor.

Another highly anticipated application of aptamers is in therapeutic applications. Research mainly investigates the replacement of antibodies by aptamers [6]. Concerning the affinity, we showed here that TBA can easily keep up with high-affinity antibodies such as the human epidermal growth factor receptor 2 (HER2)-binding cancer drug Trastuzumab (K_D_ = 660 pM) [41]. However, though both exhibit similar affinities in the 3-digit pM range, the antibody has a much longer residence time, indicated by k_OFF_ = 1.3 × 10^−4^ s^−1^. TBA, on the other hand, has an off-rate in the range of 5 × 10^−2^ s^−1^. Residence time is a crucial factor during drug development, and this comparison highlights the importance of rate determination to understand the dynamics of a potential drug in vivo. To increase residence times and specificity, antibodies are increasingly designed in bi- or even trispecific formats. If the antibody is bound by two binding sites, its off-rate decreases due to the avidity effects. This development has already been picked up for aptamer design as well [7]. Measuring off-rates of individual binding sites and the joint avidity can be challenging. First, it is difficult to mimic the mixed-target distribution in vitro, and second, the avidity can lead to affinities below the resolution limit of many methods. The switchSENSE sensor allows to immobilize different targets in a spatial- and ratio-controlled manner, enabled by a dual-color detection system. In the future, this will be applied to the combination of several thrombin-binding aptamers.

Although the aptasensor can be easily functionalized with different aptamers, the signal read-out depends on the individual sample. In the TBA example, ion binding can be resolved due to the induction of folding. Thrombin binding creates a significant signal due to the induction of folding and/or the presence of a large protein in the local surrounding of the sensing dye. Nevertheless, the detection of small molecules binding to a binding site distal to the fluorophore can require some assay optimization. If no conformational change is induced upon binding, the aptamer can be destabilized by buffer optimization or increased temperature on the sensor. Competition assays with fluorescent competitor compounds can create strong signal changes. Though requiring some knowledge of the binding site, additional fluorophores can be included site-specific at positions close to the binding site or where conformational changes are to be expected. Lastly, it can be advantageous to reverse the assay orientation and immobilize the target on the surface. Capturing the aptamer from solution by a small molecule is expected to create a very good signal.

### 3.5. Effect of Reversing the Assay Orientation

We have shown data of the aptasensor being functionalized with both the aptamer on the surface (Figure 2, Figure 3 and Figure 4) as well as the target thrombin on the surface (Figure 5). Besides creating different signal intensities by reversing the assay orientation, the setup with the target immobilized can have further applications. While a surface-immobilized aptamer can be easily subjected to all kinds of interaction tests, it is sometimes the aptamer itself that should be altered and screened, e.g., directly after the SELEX process. This can be facilitated by having the aptamer as analyte in solution. It should be noted that the rates measured with the target being immobilized could differ from the rates measured with the aptamer being immobilized. Although we used the exact same buffers and measurement scripts, for the TBA–thrombin interaction the K_D_ increased from 150 pM to 6 nM just by changing the assay orientation. Considering the broad range of pM to nM affinities reported in literature and measured with different methods [12], the assay setup is likely to have a significant influence on the values measured. However, we can neither exclude that the chemical process of thrombin conjugation to DNA alters the protein and affects the binding affinity. The optimal assay setup should be chosen based on experimental aim and include signal enhancement modifications, e.g., quenchers, if required.

## 4. Materials and Methods

### 4.1. Materials 

Human α-thrombin was purchased from Haematologic Technologies Inc. (Essex Junction, VT, USA) and suspended in TE140 buffer (10 mM Tris-HCl pH 7.4, 140 mM NaCl, 0.05% Tween20, 50 µM EDTA, 50 µM EGTA) and stored at −80 °C. The HPLC-purified DNA oligonucleotides were purchased from Ella Biotech GmbH (Martinsried, DE) and used without further purification. All oligonucleotides were suspended to a final concentration of 100 µM in TE40 (10 mM Tris-HCl pH 7.4, 40 mM NaCl, 0.05% Tween 20, 50 µM EDTA, 50 µM EGTA) buffer and stored at −20 °C.

The DNA sequences used are listed below; bold bases represent the aptamer sequence and bases in italics represent the cNL-B sequence (complementary to nanolever NL-B48 immobilized on the chip surface). Underlined bases in TBAsc represent the shuffled portion that prevents folding into a G-quadruplex. For the folding experiment (Section 2.1), TBA and TBAsc sequences were modified with a BlackBerry^®^ Quencher (BBQ-650^®^) on their 5′ end.

1. TBA: 5′(BBQ)-**GGT TGG TGT GGT TGG** TTT *ATC AGC GTT CGA TGC TTC CGA CTA ATC AGC CAT ATC AGC TTA CGA CTA*-3′;
2. TBAsc: 5′(BBQ)-**GTG TGG TGT GTG TGG** TTT *ATC AGC GTT CGA TGC TTC CGA CTA ATC AGC CAT ATC AGC TTA CGA CTA*-3′;
3. HTA: 5′-TTT **GGG TTA GGG TTA GGG TTA GGG** TTT *ATC AGC GTT CGA TGC TTC CGA CTA ATC AGC CAT ATC AGC TTA CGA CTA*-3′.

### 4.2. Covalent Conjugation of Thrombin to cNL-B

For assays requiring thrombin to be immobilized as ligand on the biochip surface, it was covalently conjugated to the 5′ end of the cNL-B48 oligonucleotide via amine chemistry in MES buffer (50 mM 2-(N-Morpholino)ethanesulfonic acid, 150 mM NaCl, pH 6.5) (amine coupling kit CK-NH_2_-7-B48, Dynamic Biosensors GmbH, Martinsried, DE). The DNA–protein conjugate was separated from non-conjugated oligonucleotide and protein by a proFIRE chromatography system (Dynamic Biosensors GmbH, Martinsried, DE) and aliquots of the pure conjugate were stored in MES buffer at −80 °C. 

### 4.3. SwitchSENSE Experiments

All experiments were performed on a DRX^2^ instrument (Dynamic Biosensors GmbH, Martinsried, DE) on standard multipurpose switchSENSE chips (MPC2-48-2-G1R1-S) using the static measurement mode. The immobilized dye was excited in the range of 600–630 nm and emission in the range of 650–685 nm was recorded. The experimental workflow was set-up using the proprietary switchBUILD software. The aptamers were diluted to a concentration of 1 µM in the relevant running buffer, heated at 82 °C for 1 min, and then cooled to 22 °C prior to use. Surface functionalization was achieved by hybridizing 500 nM of cNL-B oligonucleotide (extended by or covalently linked to the respective ligand) to the single-stranded pre-immobilized NL-B48 oligo on the chip surface in auxiliary buffer TE40. Association of analyte of interest (ions, thrombin, aptamers) and dissociation in running buffer were performed with a flow rate of 2000 µL/min at 25 °C, except where noted otherwise. Surface regenerations were only performed at the beginning of a measurement set, no regenerations were carried out in between different concentrations of one set. The folding measurements were carried out in a background of Tris50 buffer (50 mM Tris-HCl pH 7.4, 0.005% Tween 20), and the kinetic measurements in TE140-XCl buffer (10 mM Tris-HCl pH 7.4, 140 mM XCl, 0.05% Tween 20, 50 µM EDTA, 50 µM EGTA, with XCl representing different salt species: KCl, NaCl, NH_4_Cl, LiCl). 

### 4.4. Data Analysis

Fluorescence recorded was referenced with the control TBAsc in Figure 2 (raw data are shown in Supplementary Materials Figure S4); all other figures show raw data normalized to the baseline before analyte injection. The fluorescence traces were analyzed with the switchANALYSIS software (Dynamic Biosensors GmbH, DE) by fitting all association and dissociation curves of one dataset simultaneously (global fit) with a single-exponential fit model. Measurements for Figure 2 and Figure 3 were performed in triplicates, and values reported represent the mean k_ON_ and k_OFF_ of three datasets, leading to an average K_D_ by:

K_D_ = k_OFF,avg_/k_ON,avg_ (1)

±standard deviation with error propagation:

SD = K_D_ · ((δk_OFF_/k_OFF_)^2^ + (δk_ON_/k_ON_)^2^)^1/2^ (2)

Measurements for Figure 5B were performed in triplicates and recorded data were averaged before applying the global single-exponential fit to derive k_ON_ and k_OFF_. Individual datasets of triplicates are shown in Supplementary Materials Figures S5 and S6.

The folding rate at highest salt concentration applied was deduced from the time constant τ of mono-exponential fits by:

k_F_ = 1/τ_ON_ (3)

Surface saturation levels were calculated based on the measured rates in the Kinetics tool of the switchBUILD software (Dynamic Biosensors GmbH, DE).

Rate plot in Figure 2 and Supplementary Materials Figure S1 were plotted via www.affinity-avidity.com and the labeling was adjusted to the color code.

## 5. Conclusions

In this work, we presented a rapid, sensitive, and selective aptamer-ion/protein assay based on an aptasensor. The significance of this work lies in the easy transfer of the assay from TBA–thrombin interaction to any aptamer-–target pair. The DNA-based switchSENSE technology allows quasi modification-free immobilization of low amounts of aptamer on the biosensor via hybridization. High-information content data of folding, unfolding, association, and dissociation rates enabled conclusions about ion-induced aptamer folding and a protein-induced fit mechanism.

The type-of-cation-dependent k_ON_ rates are direct evidence as to why detection limits and affinities achieved for TBA–thrombin strongly vary in literature. A broad dynamic range from low pM to high nM suggests a future diagnostic application of the developed aptasensor. Furthermore, the resolution of highly dynamic target association and dissociation rates helps to understand residence times in therapeutic applications. In conclusion, the described aptasensor can contribute significantly to the areas of aptamer development and characterization, aptamer-based diagnostics, as well as apta-therapeutics.

## Figures and Tables

**Table 1:** Summary of ion-TBA kinetic parameters in 50 mM Tris buffer, observed by the conformational change they induce. Rates of ion binding were obtained by global mono-exponential fits. The ion dissociation rate k_OFF_ represents the minimum rate to allow the observed unfolding rate k_U_. The folding rate k_F_ was the observed rate at highest salt concentration tested. Values shown represent the mean (±standard deviation, SD) of three independent experiments.

Ion	k_ON_ (M^−1^s^−1^)	k_OFF_ = k_U_ (10^−1^ s^−1^)	K_D_ (mM)	k_F_ (s^−1^)	K_F_ (No Unit)
NH_4_^+^	2.96 ± 0.8	3.07 ± 0.3	104 ± 40	1.18 ± 0.23	3.84 ± 0.8
K^+^	15.2 ± 1.7	1.20 ± 0.01	7.89 ± 1.3	1.26 ± 0.14	10.4 ± 1.1
Na^+^	-	-	-	-	-
Li^+^	-	-	-	-	-

k_ON_ = rate constant of ion binding to TBA (M^−^^1^s^−^^1^); k_OFF_ = minimum rate constant of ion dissociation from TBA (s^−^^1^); K_D_ = equilibrium dissociation constant of ion (M) calculated by K_D_ = k_OFF_/k_ON_; k_F_ = observed rate of TBA folding (s^−^^1^); k_U_ = rate constant of TBA unfolding (s^−^^1^); K_F_ = ratio of TBA folding versus unfolding calculated by K_F_ = k_F_/k_U_.

**Table 2:** Summary of thrombin–TBA kinetic parameters in indicated buffers. Data represent the mean (±standard deviation, SD) of three independent experiments.

Buffer	k_ON_ (10^7^ M^−1^s^−1^)	k_OFF_ (10^−2^ s^−1^)	K_D_ (nM)
TE140-KCl	38 ± 1.3	5.8 ± 0.1	0.15 ± 0.01
TE140-NH_4_Cl	17 ± 1.0	4.8 ± 0.1	0.28 ± 0.02
TE140-NaCl	3.3 ± 0.2	4.3 ± 0.1	1.31 ± 0.12
TE140-LiCl	0.03 ± 0.01	6.3 ± 0.4	245 ± 94

## Supplementary Materials

The following figures are available online, Figure S1: Thrombin kinetics in TE140-KCl at different flow rates, Figure S2: Thrombin association at low ionic strength (50 mM Tris with no salts added), Figure S3: Comparison of quenching amplitudes with surface saturation. Section S2: Explanation of the switchSENSE detection mode FPS. Figure S4: Raw data of TBA (triplicates) and TBAsc folding experiments, Figure S5: Triplicates of thrombin kinetics experiments, Figure S6: Triplicates of reversed assay orientation kinetics experiment.
