# Supplementary material for: A DNA-Based Biosensor Assay for the Kinetic Characterization of Ion-Dependent Aptamer Folding and Protein Binding
Source: Molecules. 2019 Aug 8;24(16):2877. doi: 10.3390/molecules24162877 (PMC6718989; doi:10.3390/molecules24162877)
Supplement: Supplementary file 1 [file molecules-24-02877-s001.pdf]

Supplementary Materials:

A DNA-Based Biosensor Assay for the Kinetic Characterization of Ion-Dependent Aptamer Folding and Protein Binding

Irene Ponzo, Friederike M. Möller, Herwin Daub and Nena Matscheko

Dynamic Biosensors GmbH, Lochhamer Str. 15, 82152 Martinsried, Germany

Content:

Figure S1: Thrombin kinetics in TE140-KCl at different flow rates.

Figure S2: Thrombin association at low ionic strength (50 mM Tris with no salts added).

Figure S3: Comparison of quenching amplitudes with surface saturation.

Section S1: Explanation switchSENSE detection mode FPS.

Figure S4: Raw data of TBA (triplicates) and TBAsc folding experiments.

Figure S5: Triplicates of thrombin kinetics experiments.

Figure S6: Triplicates of reversed assay orientation kinetics experiment.

Section S1

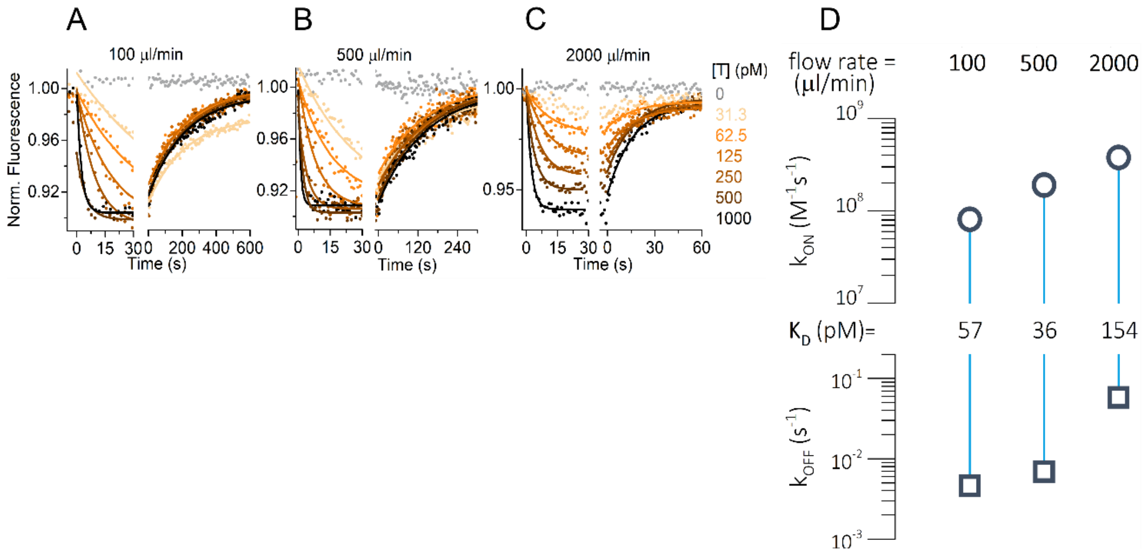

Figure S1. Thrombin kinetics in TE140-KCl at different flow rates. Interaction of thrombin at specified concentrations with surface-immobilized TBA carried out at **A**) 100 µl/min, **B**) 500 µl/min, **C**) 2000 µl/min. **D**) Rate plot of values obtained by global mono-exponential fits from **A**)-**C**). Lower flow rates result in reduced on-rate and reduced off-rate. Increase of the flow rate reduces measurement artifacts such as mass transport limitation or rebinding.

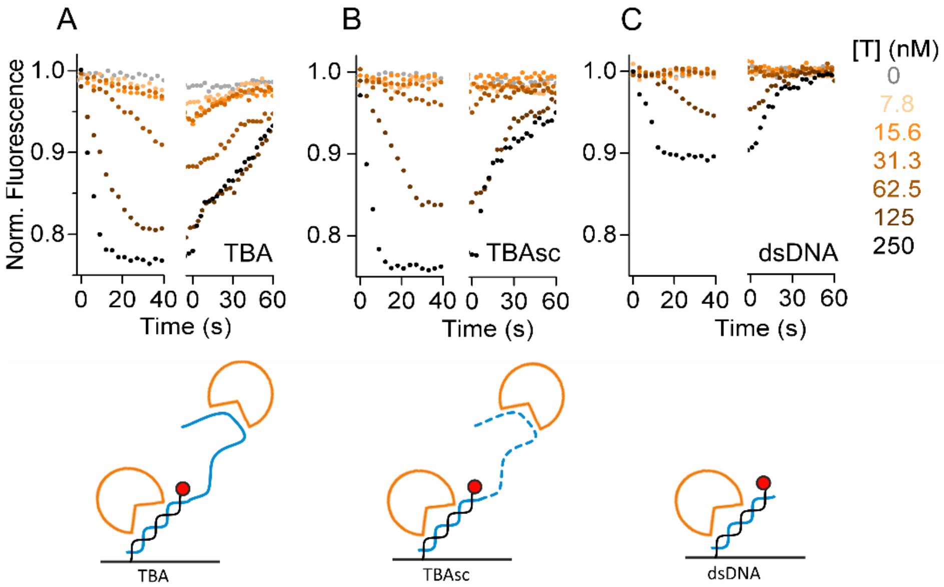

Figure S2. Thrombin association at low ionic strength (50 mM Tris with no salts added). **A**) Thrombin binding to both TBA and **B**) TBAsc is observed at ≥ 62.5 nM thrombin. **C**) Non-specific thrombin binding to dsDNA is observed at ≥ 125 nM thrombin.

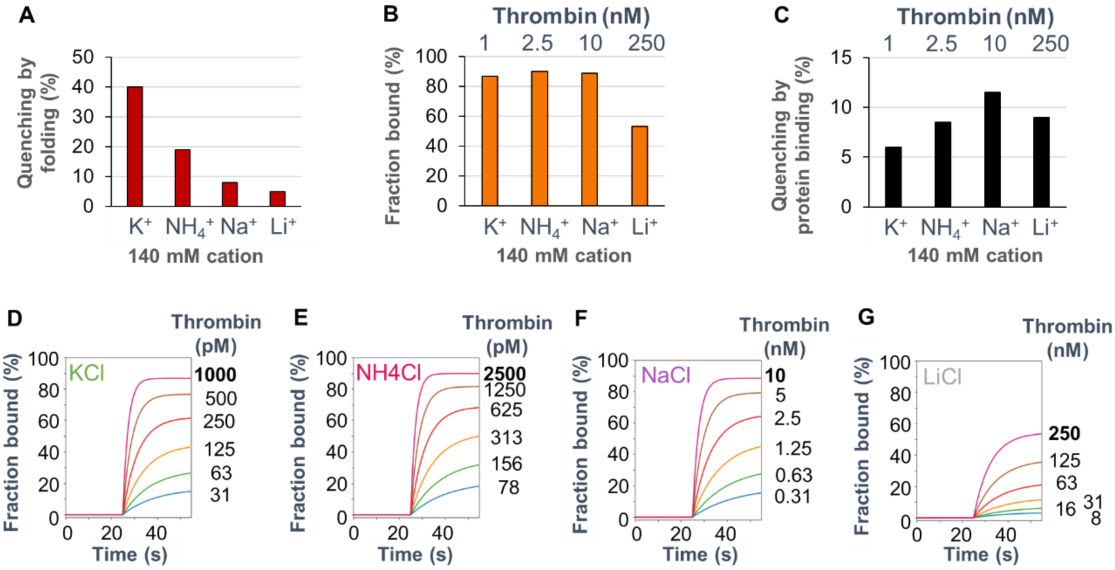

Figure S3. Comparison of quenching amplitudes with surface saturation. **A**) Fluorescence quenching (%) induced by 140 mM cation derived from Figure 2. K+ reached signal saturation at 75 mM, therefore the same quenching was assumed for 140 mM. **B**) Plot of the calculated fraction of TBA bound by thrombin (%) at the highest protein concentration tested in the respective buffers. Fractions bound were derived from the plots in **D-G**). **C**) Fluorescence quenching (%) induced by thrombin binding. Values were extracted from Figure 3. Total quenching is lower than in A) since quenching was achieved by guanine instead of BBQ. **D-G**) The cation-dependent fractions bound (%) of TBA at different thrombin concentrations, plotted by the switchBUILD software based on the kinetic rates determined in Figure 3.

Section S2: Explanation switchSENSE detection mode FPS

switchSENSE features two complementary measurement modes. In static measurement mode (Fluorescence Proximity Sensing, FPS) the DNA strands are repelled from the surface (constant voltage, $V_{attractive} = V_{repulsive} = -0.1$ V). The fluorophore attached to the distal end of the DNA therefore remains at maximum distance from the gold electrode. For signal detection of biomolecular interactions, the fluorescence intensity of the dye is read out. It changes its fluorescence emission upon altered static or collisional quenching by complex formation of ligand and analyte. The fluorescence signal change is proportional to the surface bound analytes.

For more information please visit Dynamic Biosensors' website
https://www.dynamic-biosensors.com/switchsense/

Section S3: Source data of figures indicated

Figure S4 to Figure 2: raw data of TBA (triplicates) and TBAsc folding experiments in K^+, $NH4^+$, Na^+, Li^+
Figure S5 to Figure 3: triplicates of thrombin kinetics experiments in K^+, Na^+, $NH4^+$, Li^+
Figure S6 to Figure 5: triplicates of reversed assay orientation kinetics experiment in K^+

Figure S4

Figure 2B: Folding with K+

TBA

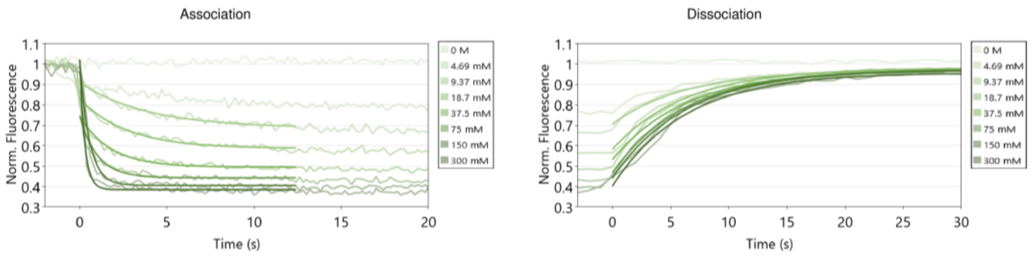

k_{ON} = 11.08 ± 0.34 $M^{-1}\cdot s^{-1}$ k_{OFF} = 1.59 ± 0.03 E-1 s^{-1} K_D = 14.3 ± 0.5 mM

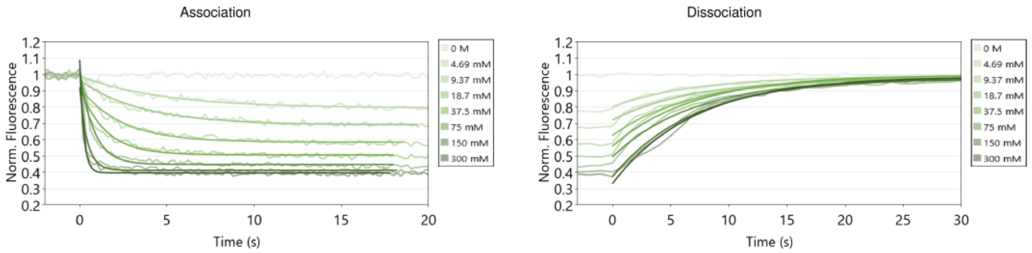

k_{ON} = 14.59 ± 0.4 $M^{-1}\cdot s^{-1}$ k_{OFF} = 1.43 ± 0.02 E-1 s^{-1} K_D = 9.83 ± 0.29 mM

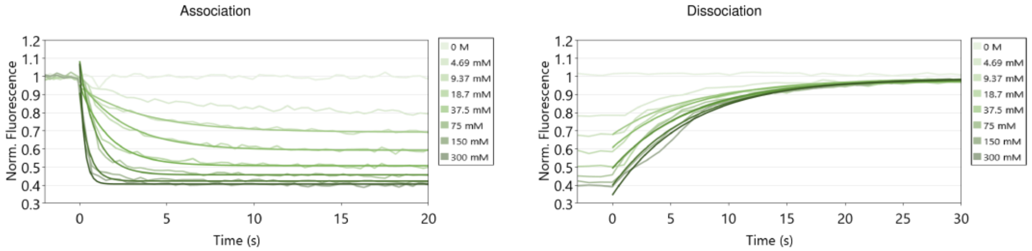

k_{ON} = 12.2 ± 0.29 $M^{-1}\cdot s^{-1}$ k_{OFF} = 1.50 ± 0.02 E-1 s^{-1} K_D = 12.3 ± 0.3 mM

TBAsc

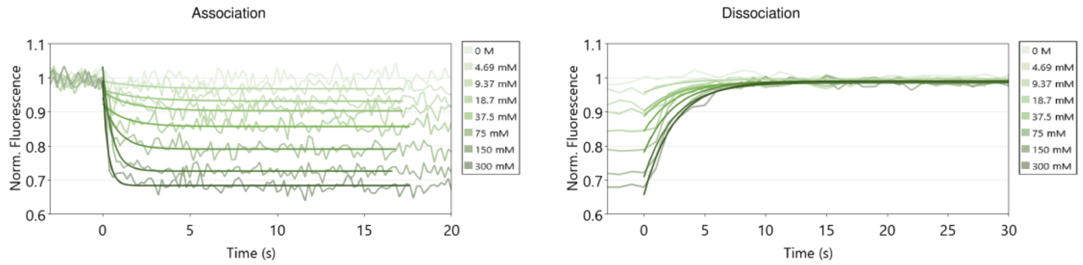

k_{ON} = 10.15 ± 0.81 $M^{-1}\cdot s^{-1}$ k_{OFF} = 4.04 ± 0.12 E-1 s^{-1} K_D = 39.8 ± 3.4 mM

Figure S4
Figure 2C: Folding with NH4+

TBA

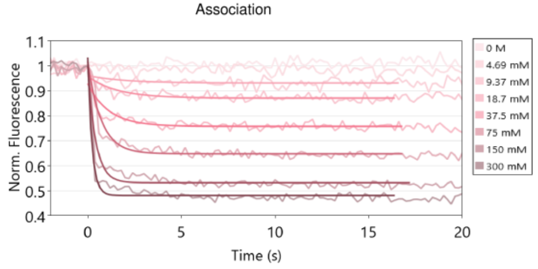

k_{ON} = 13.17 ± 0.6 $M^{-1} \cdot s^{-1}$

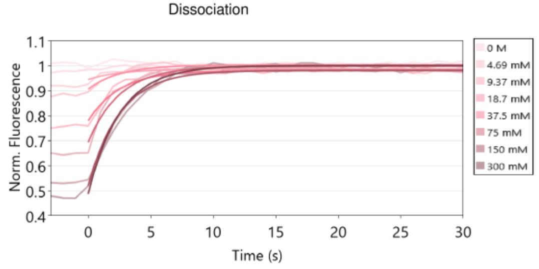

k_{OFF} = 3.94 ± 0.08 E-1 s^{-1}

K_D = 29.9 ± 1.5 mM

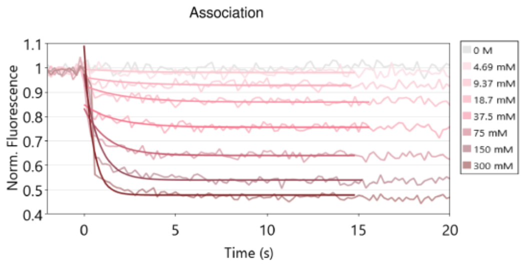

k_{ON} = 5.93 ± 0.31 $M^{-1} \cdot s^{-1}$

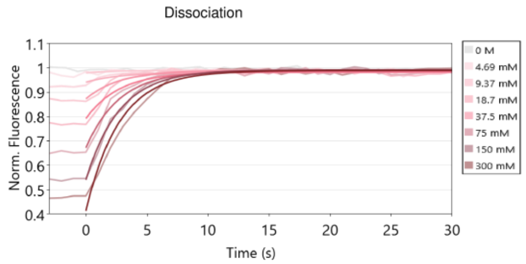

k_{OFF} = 3.66 ± 0.08 E-1 s^{-1}

K_D = 61.8 ± 3.5 mM

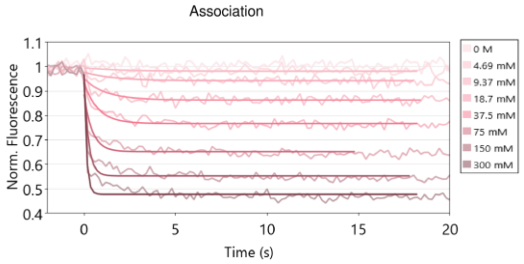

k_{ON} = 23.98 ± 1.42 $M^{-1} \cdot s^{-1}$

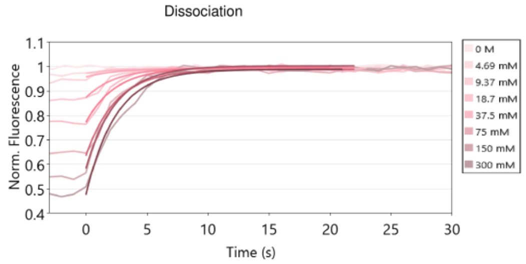

k_{OFF} = 4.10 ± 0.11 E-1 s^{-1}

K_D = 17.1 ± 1.1 mM

TBAsc

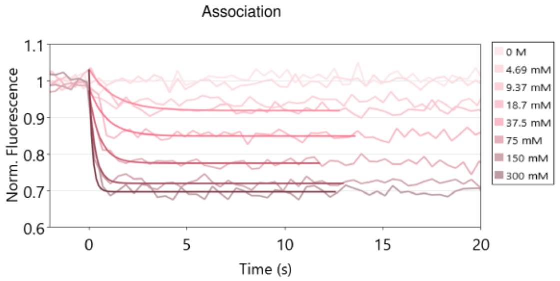

k_{ON} = 17.89 ± 1.64 $M^{-1} \cdot s^{-1}$

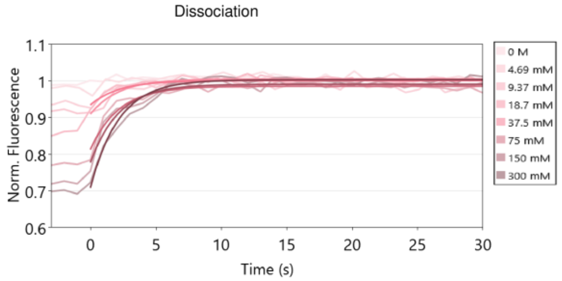

k_{OFF} = 4.64 ± 0.19 E-1 s^{-1}

K_D = 25.9 ± 2.6 mM

Figure S4
Figure 2D: Folding with Na+

TBA

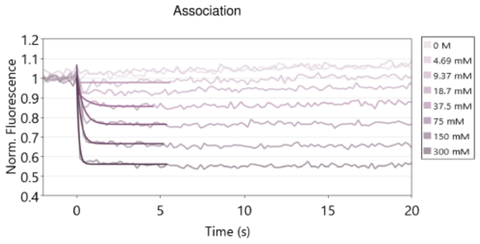

k_{ON} = 24.36 ± 2.25 $M^{-1} \cdot s^{-1}$

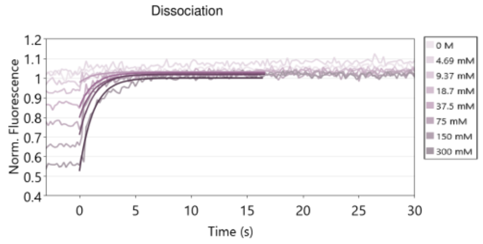

k_{OFF} = 8.15 ± 0.24 E-1 s^{-1}

K_D = 33.5 ± 3.2 mM

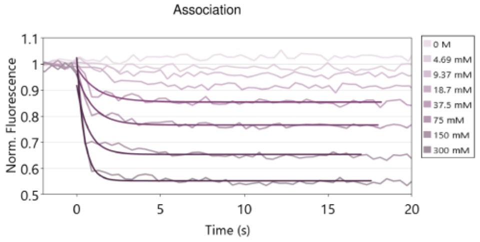

k_{ON} = 5.44 ± 0.43 $M^{-1} \cdot s^{-1}$

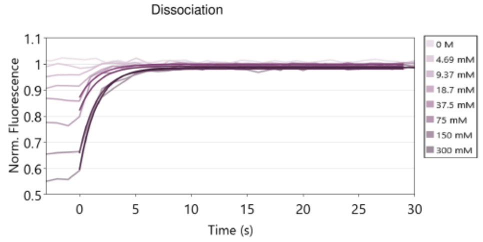

k_{OFF} = 6.33 ± 0.19 E-1 s^{-1}

K_D = 116 ± 10 mM

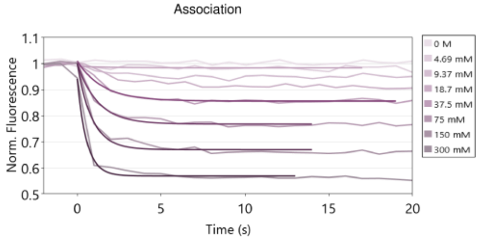

k_{ON} = 4.33 ± 0.37 $M^{-1} \cdot s^{-1}$

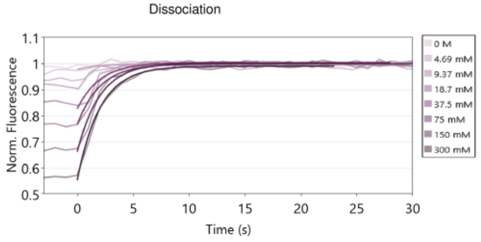

k_{OFF} = 4.93 ± 0.16 E-1 s^{-1}

K_D = 114 ± 10 mM

TBAsc

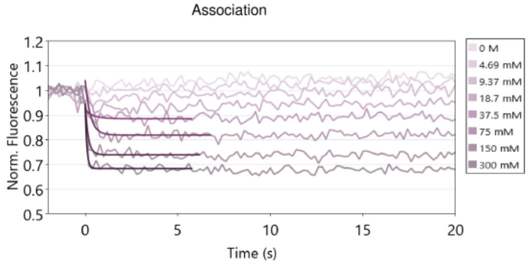

k_{ON} = 33.52 ± 4.78 $M^{-1} \cdot s^{-1}$

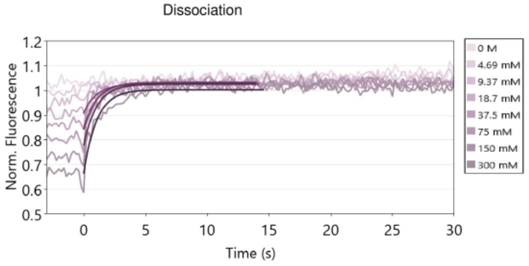

k_{OFF} = 9.53 ± 0.52 E-1 s^{-1}

K_D = 28.4 ± 4.3 mM

Figure S4
Figure 2E: Folding with Li+

TBA

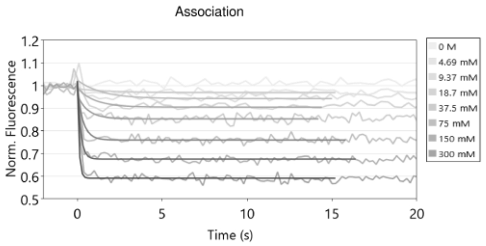

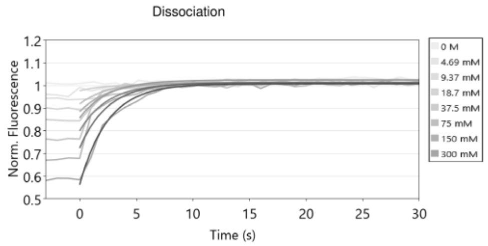

k_{ON} = 31.89 ± 2.59 $M^{-1}·s^{-1}$

k_{OFF} = 4.07 ± 0.11 E-1 s^{-1}

K_D = 12.8 ± 1.1 mM

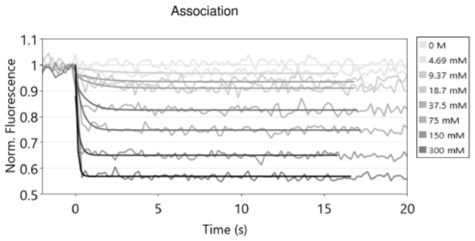

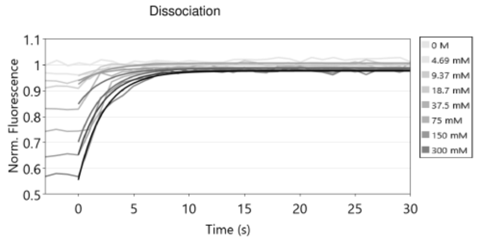

k_{ON} = 27.97 ± 2.19 $M^{-1}·s^{-1}$

k_{OFF} = 4.38 ± 0.11 E-1 s^{-1}

K_D = 15.6 ± 1.3 mM

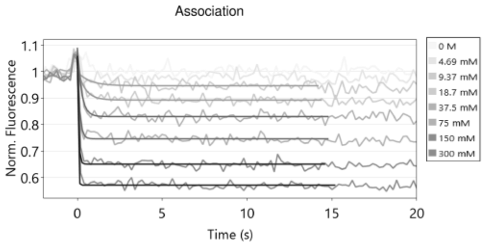

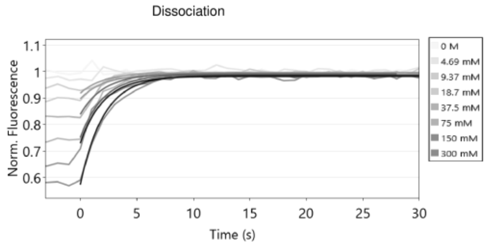

k_{ON} = 94.21 ± 7.36 $M^{-1}·s^{-1}$

k_{OFF} = 4.86 ± 0.14 E-1 s^{-1}

K_D = 5.16 ± 0.43 mM

TBAsc

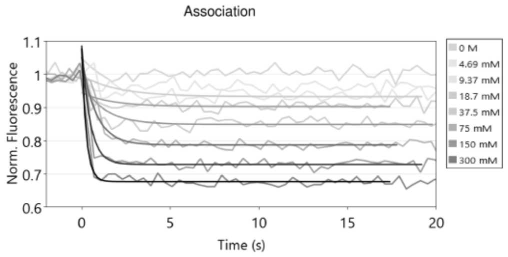

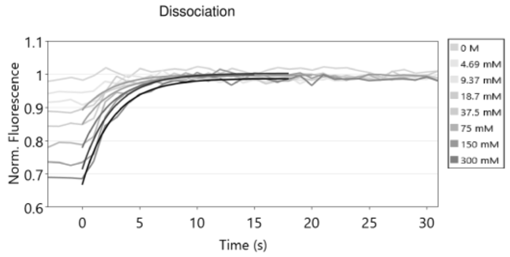

k_{ON} = 11.68 ± 0.94 $M^{-1}·s^{-1}$

k_{OFF} = 3.74 ± 0.17 E-1 s^{-1}

K_D = 32.0 ± 3.0 mM

Figure S5
Figure 3B: Thrombin binding in TE140-KCl

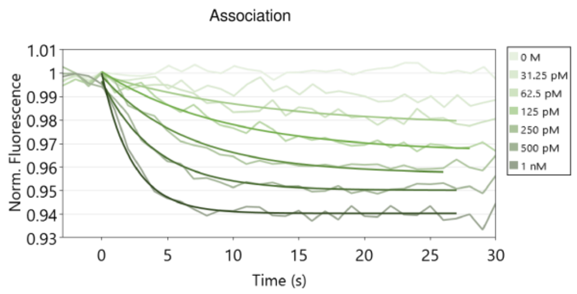

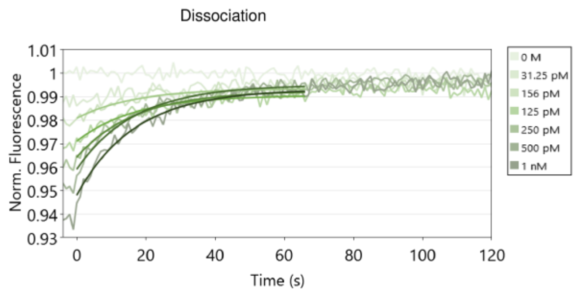

k_{ON} = 3.72 ± 0.20 E+8 $M^{-1} \cdot s^{-1}$

k_{OFF} = 5.99 ± 0.19 E-2 s^{-1}

K_D = 161 ± 10 pM

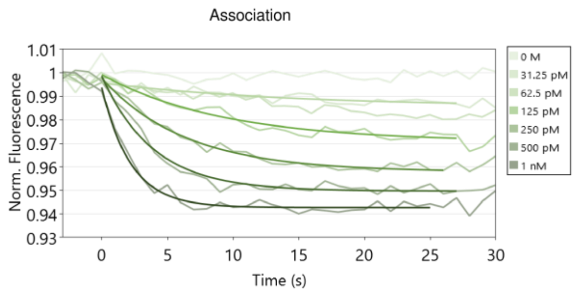

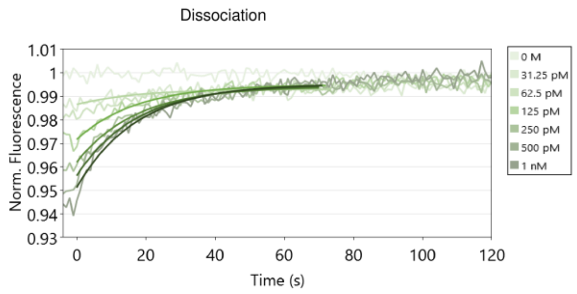

k_{ON} = 3.97 ± 0.20 E+8 $M^{-1} \cdot s^{-1}$

k_{OFF} = 5.82 ± 0.18 E-2 s^{-1}

K_D = 147 ± 9 pM

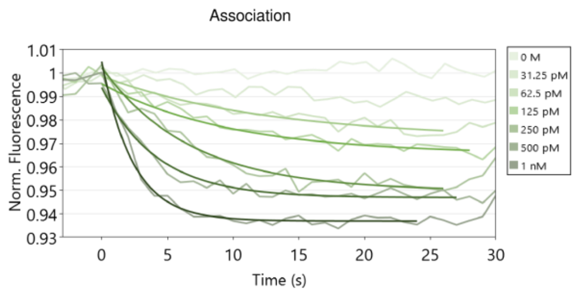

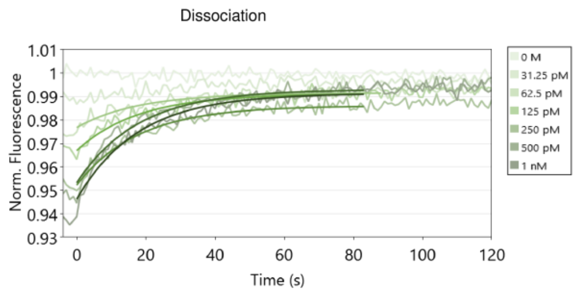

k_{ON} = 3.66 ± 0.18 E+8 $M^{-1} \cdot s^{-1}$

k_{OFF} = 5.66 ± 0.17 E-2 s^{-1}

K_D = 155 ± 9 pM

Figure S5

Figure 3C: Thrombin binding in TE140-NaCl

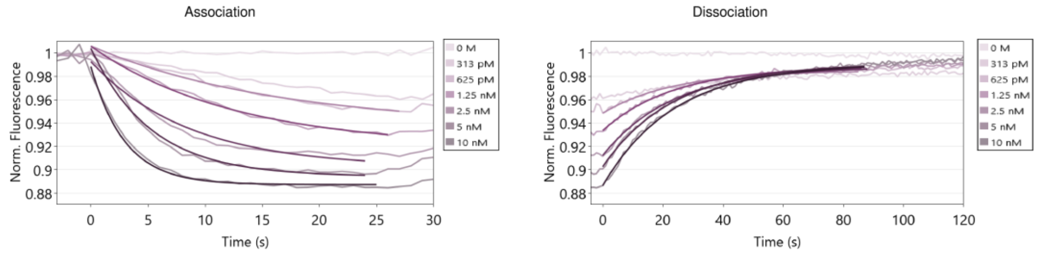

k_{ON} = 2.95 ± 0.11 E+7 $M^{-1} \cdot s^{-1}$

k_{OFF} = 4.25 ± 0.04 E-2 s^{-1}

K_D = 1.44 ± 0.05 nM

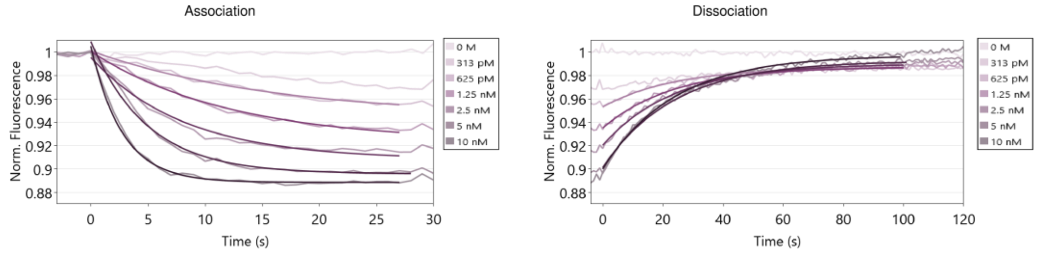

k_{ON} = 3.24 ± 0.09 E+7 $M^{-1} \cdot s^{-1}$

k_{OFF} = 4.21 ± 0.04 E-2 s^{-1}

K_D = 1.3 ± 0.0 nM

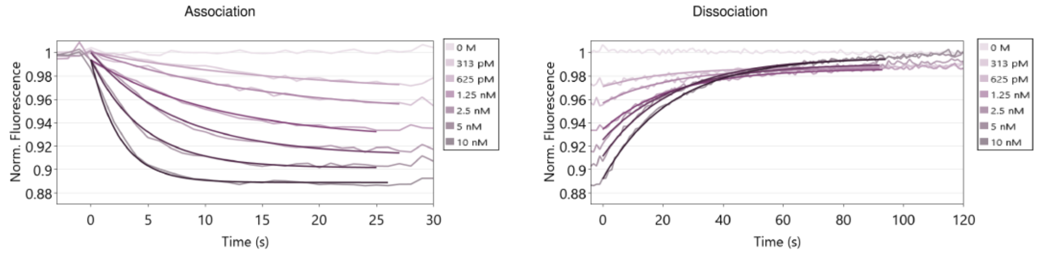

k_{ON} = 3.55 ± 0.09 E+7 $M^{-1} \cdot s^{-1}$

k_{OFF} = 4.39 ± 0.04 E-2 s^{-1}

K_D = 1.24 ± 0.03 nM

Figure S5
Figure 3D: Thrombin binding in TE140-NH4Cl

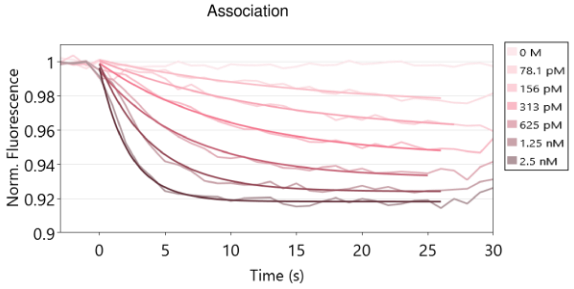

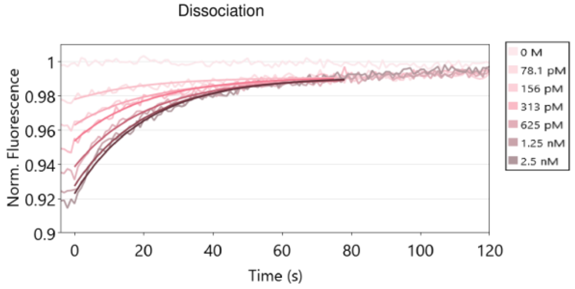

k_{ON} = 1.60 ± 0.06 E+8 $M^{-1} \cdot s^{-1}$ k_{OFF} = 4.88 ± 0.09 E-2 s^{-1} K_D = 306 ± 12 pM

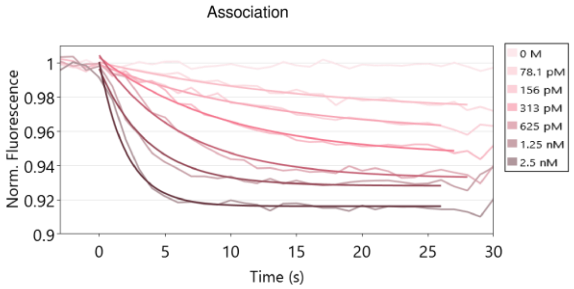

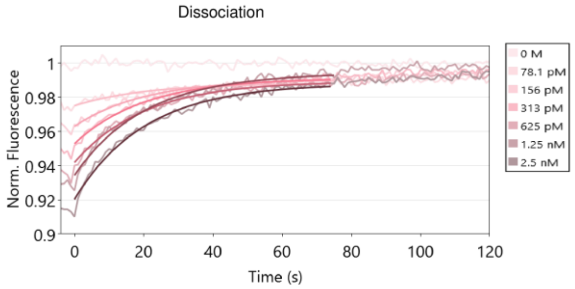

k_{ON} = 1.74 ± 0.07 E+8 $M^{-1} \cdot s^{-1}$ k_{OFF} = 4.94 ± 0.10 E-2 s^{-1} K_D = 284 ± 13 pM

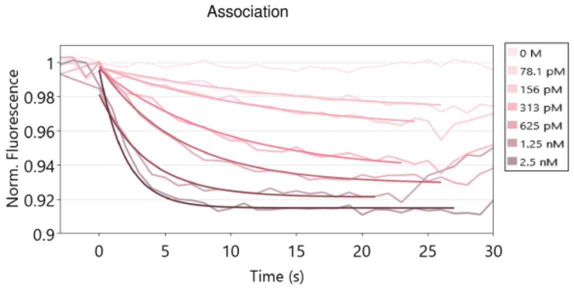

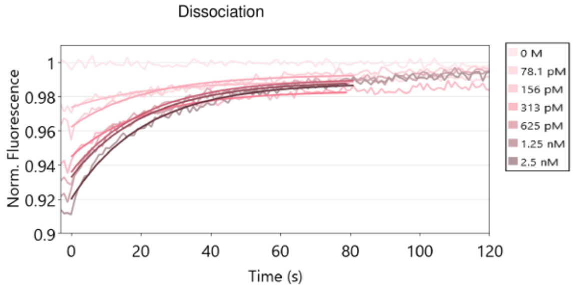

k_{ON} = 1.84 ± 0.08 E+8 $M^{-1} \cdot s^{-1}$ k_{OFF} = 4.79 ± 0.09 E-2 s^{-1} K_D = 260 ± 12 pM

Figure S5
Figure 3E: Thrombin binding in TE140-LiCl

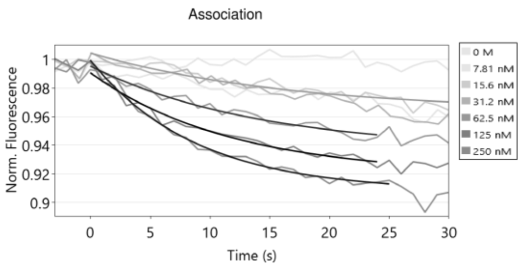

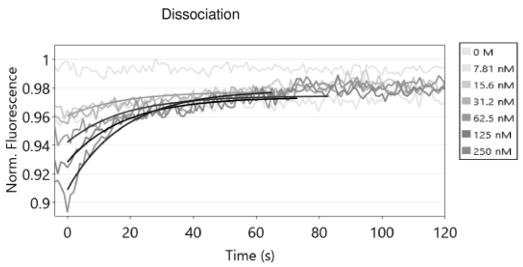

k_{ON} = 2.28 ± 0.34 E+5 $M^{-1} \cdot s^{-1}$ k_{OFF} = 5.64 ± 0.30 E-2 s^{-1} K_D = 248 ± 39 nM

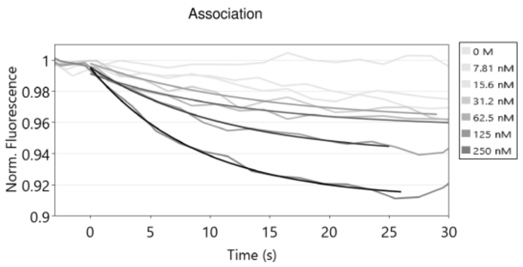

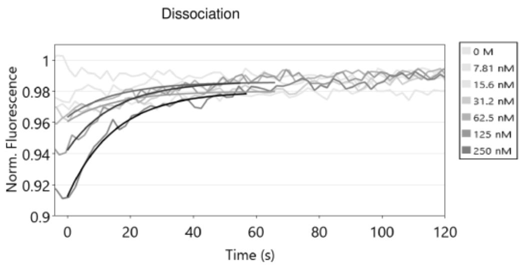

k_{ON} = 1.74 ± 0.36 E+5 $M^{-1} \cdot s^{-1}$ k_{OFF} = 6.66 ± 0.37 E-2 s^{-1} K_D = 381 ± 83 nM

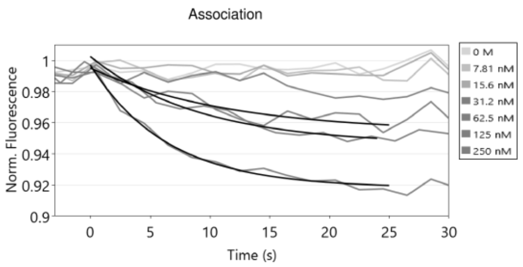

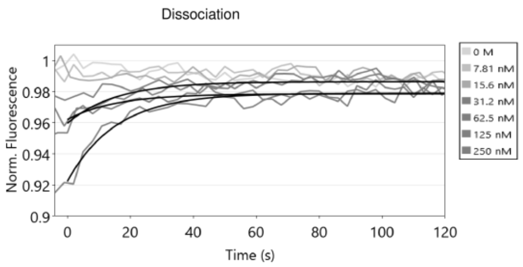

k_{ON} = 3.64 ± 0.74 E+5 $M^{-1} \cdot s^{-1}$ k_{OFF} = 6.49 ± 0.41 E-2 s^{-1} K_D = 179 ± 38 nM

Figure S6
Figure 5B: TBA binding to Thrombin in TE140-KCl

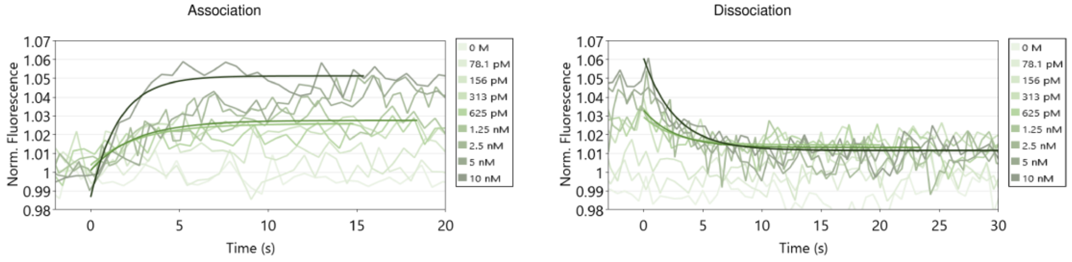

k_{ON} = 2.61 ± 1.00 E+7 $M^{-1} \cdot s^{-1}$

k_{OFF} = 3.71 ± 0.55 E-1 s^{-1}

K_D = 14.2 ± 5.8 nM

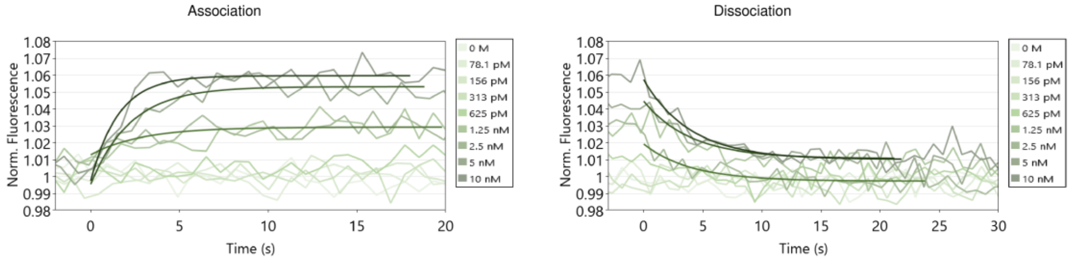

k_{ON} = 3.91 ± 0.86 E+7 $M^{-1} \cdot s^{-1}$

k_{OFF} = 2.51 ± 0.34 E-1 s^{-1}

K_D = 6.42 ± 1.65 nM

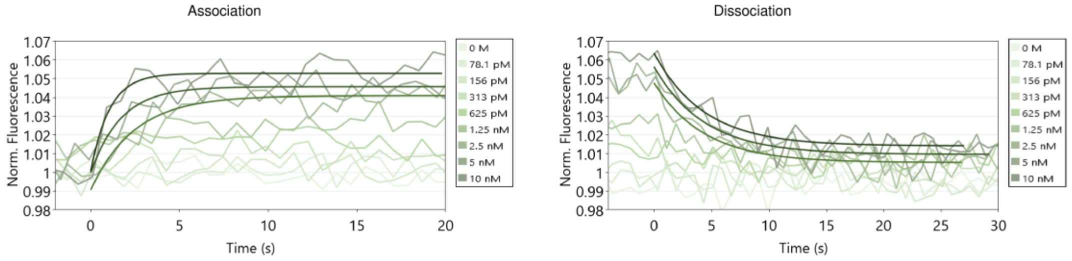

k_{ON} = 7.18 ± 1.54 E+7 $M^{-1} \cdot s^{-1}$

k_{OFF} = 2.30 ± 0.23 E-1 s^{-1}

K_D = 3.2 ± 0.8 nM
